# Assessment of Pathogenic Potential, Virulent Genes Profile, and Antibiotic Susceptibility of *Proteus mirabilis* from Urinary Tract Infection

**DOI:** 10.1155/2020/1231807

**Published:** 2020-02-07

**Authors:** Emad I. Hussein, Khalid Al-Batayneh, Majed M. Masadeh, Fatina W. Dahadhah, Mazhar Salim Al Zoubi, Alaa A. Aljabali, Karem H. Alzoubi

**Affiliations:** ^1^Department of Biological Sciences, Yarmouk University, Irbid 21163, Jordan; ^2^Department of Food Science and Human Nutrition, A'Sharqiyah University, Ibra, Oman; ^3^Department of Pharmaceutical Technology, Jordan University of Science and Technology, Irbid 22110, Jordan; ^4^Department of Basic Medical Sciences, Faculty of Medicine, Yarmouk University, Irbid 21163, Jordan; ^5^Department of Pharmaceutical Sciences, Yarmouk University, Irbid 21163, Jordan; ^6^Department of Clinical Pharmacy, Jordan University of Science and Technology, Irbid 22110, Jordan

## Abstract

*Proteus mirabilis* is the third most common bacterium that can cause complicated UTI, especially in catheterized patients. Urovirulence genes of *P. mirabilis* strains are poorly identified among UTI patients. The aims of the present study were to determine the prevalence of the uropathogenic *P. mirabilis* strains isolated from UTI patients by the detection of several *P. mirabilis* virulence genes and to characterize the antibiotic susceptibility profile of *P. mirabilis* isolates. *P. mirabilis* isolates were collected from urine specimens of patients suffering from UTI. Virulence genes in *P. mirabilis*, namely, *hpmA*, *hpmB*, *rsbA*, *luxS*, *ureC1*, *hlyA*, *rpoA*, *atfA*, *atfC*, *mrpA*, and *pm1* were detected in the isolates via PCR detection method. All *P. mirabilis* virulence genes were detected in more than 90% of the isolates except *hlyA* gene, which was detected in only 23.8% of the isolates. The rate of susceptibility for ceftriaxone was 96.8%, followed by norfloxacin (82.5%), gentamicin (71.4%), ciprofloxacin (69.8%), cephalexin (52.4%), nalidixic acid (42.9%), sulfamethoxazole (39.7%), ampicillin (36.5%), and nitrofurantoin (3.2%). Significant associations (*P* < 0.05) were detected between antimicrobial susceptibility of each of the following antibiotics and the presence virulence genes. Cephalexin antimicrobial susceptibility was significantly associated with the presence each of ureC1 and atfC. Sulfamethoxazole antimicrobial susceptibility was significantly associated with the presence atfA. Ceftriaxone antimicrobial susceptibility was significantly associated with the presence each of *hpmA*, *ureC1*, *rpoA*, *atfC*, *mrpA*, and *pm1*. Nitrofurantoin antimicrobial susceptibility was significantly associated with the presence each of *hpmA*, *ureC1*, *rpoA*, *atfA*, *atfC*, *mrpA*, and *pm1*. In conclusion, an association between the presence of urovirulence genes of *P. mirabilis* and increasing *P. mirabilis* resistance to antimicrobials has been demonstrated.

## 1. Introduction

*Proteus mirabilis* is one of the most common Gram-negative bacteria that can cause UTIs. Bacteriuria, kidney stones, catheter obstruction, acute pyelonephritis, and fever can be developed by *P. mirabilis* [1]. In fact, *P. mirabilis* strains are responsible for the majority of complicated urinary tract infections [1]. *P. mirabilis* is becoming resistant to antibiotics commonly used in the treatment of UTI [2]. The bacterium was shown to be highly sensitive to streptomycin (100%), erythromycin (85%), and sparfloxacin (75%), whereas it showed high resistance to amoxicillin (100%), tetracycline (95%), and cefuroxime (80%). Isolated *Proteus mirabilis* has shown multiple drug-resistance ability to the used antibiotics [3].

*P. mirabilis* encodes many virulence genes involved in infection [4, 5]. Urease (*ureC1*) is a virulence gene that is important in *P. mirabilis* pathogenesis. This enzyme catalyzes the kidney and bladder stone formation or blocks indwelling urinary catheters [6]. Urease is required for urolithiasis, where it contributes in hydrolyzing urea to release ammonia, thereby increasing urinary pH, resulting in precipitation of calcium and magnesium compounds, and urinary stone formation [7]. The alteration of pH is important in catheter colonization of *P. mirabilis*; facilitating the bacterial adherence and biofilm formation [4, 8]. Another group of virulence genes is the quorum sensing (*luxS*, and *rsbA*). The *luxS* gene produces signal that is used to sense the interaction of species and its cell density in a polymicrobial community that plays critical roles in the virulence genes regulation [5]. The *rsbA* gene expresses a histidine-containing phosphotransmitter of the bacterial two-component signaling system. This gene regulates the swarming manner, which encodes a sensory and act as a protein sensor of environmental circumstances [5]. Subsequently, *rsbA* facilitated biofilm and extracellular polysaccharide formation [4]. The Mannose-resistant/*Proteus-*like fimbriae (MR/P) are related to bladder and kidney infection [9]. The *mr/p* gene cluster comprised two transcripts: *mrp*ABCDEFGHJ (operon) and *mrpI*. The main structural subunit is *mrpA* protein, that is required at the first step of infection, including formation of clusters, and is important for wild-type levels of bladder colonization at the following steps [10]. The hemolytic activity of *P. mirabilis* is related to hemolysin *hpmA* and *hpmB* proteins. *hpmA* is mainly responsible for tissue damage, and *hpmA* becomes active after cleavage of its N-terminal peptide [6]. The activation and transportation of *hpmA* depend on *hpmB* hemolysin [6]. Previous studies suggest that hemolysin plays a critical role in UTI caused by *P. mirabilis*, which contributes to the potential urovirulence of *P. mirabilis* [11]. Another type of hemolysin proteins that *Proteus* species can encode and express is *hlyA*, and *Proteus* can encode *hlyA* gene similar to that virulence gene of *E. coli* [11].

Thus, there are many virulence genes that assist survival of *P. mirabilis* within the urinary system such as urease, hemolysin, fimbriae, and flagella [12]. However, *P. mirabilis* strains differ in the range and expression levels of virulence genes that can affect growth of bacteria and persistence within the urinary tract. A number of studies have investigated the virulence characteristics of *P. mirabilis* and mechanisms involved in pathogenesis of UTI to identify the range of *P. mirabilis* virulence genes and their prevalence among *P. mirabilis* isolates [4]. In the present study, *P. mirabilis* isolates involving in human UTI were characterized to identify virulence gene markers in an effort to explore strategies involved in *P. mirabilis* pathogenesis and antibiotics susceptibility.

## 2. Materials and Methods

### 2.1. *P. mirabilis* Isolates

*P. mirabilis* isolates were collected from urine samples of patients who had UTIs and significant bacterial counts (>10^5^ CFUs/mL) as per institutional ethics committee approval. Pure cultures were stored at −80°C in Luria Bertani (LB) broth with 10% glycerol [13]. Samples were collected from July to December 2017 from Jordanian Royal Medical Services. *P. mirabilis* was identified as per standard diagnostic criteria using its known characteristic of swarming motility and inability to metabolize lactose on a MacConkey agar plate [8].

### 2.2. Antimicrobial Susceptibility Testing

The following antimicrobials were used in the current study: ciprofloxacin (5 *μ*g, Hikma Pharmaceutical, Jordan), cephalexin (30 *μ*g, Dar Al Dawa, Jordan), nalidixic acid (30 *μ*g, Hikma Pharmaceutical, Jordan), sulfamethoxazole (25 *μ*g, Dar Al Dawa, Jordan), ceftriaxone (30 *μ*g, Pfizer, USA), nitrofurantoin (300 *μ*g, Jordan River Pharmaceutical Industries, Amman), norfloxacin (10 *μ*g, Amman Pharmaceutical industries, Jordan), ampicillin (10 *μ*g, Jordan Veterinary and Agriculture Medical Industrial Company, Amman), and gentamicin (10 *μ*g, Hikma Pharmaceutical, Jordan).

Kirby-Bauer disk diffusion method was used to determine the susceptibility of bacteria to antibiotic agents. Bacterial colonies were transferred from the nutrient agar plate into bottles containing NaCl 0.9% to obtain bacterial density of 1.5 × 10^8^ organisms per milliliter as determined by McFarland standard scale number 0.5 [14]. The cultures were uniformly streaked onto fresh Mueller Hinton agar plates using sterile cotton swabs. The plates were allowed to dry-off briefly, and then the discs of different antimicrobials were mounted onto the surface of the streaked inoculums. The plates were incubated at 37°C for 24 hours. Then, the culture plates were examined for inhibition. The zones of growth inhibition were measured using a meter rule described previously [15].

### 2.3. Extraction of Genomic DNA

For all isolates, several bacterial colonies were inoculated in 5 mL Luria Bertani (LB) broth media followed by incubation for 18 hours at 37°C. 1.5 mL of overnight Luria broth bacterial growth culture was subjected to DNA extraction using genomic DNA isolation kit OMEGA bacterial DNA purification kit [16]. Isolated DNA samples were stored at −20°C till later use.

### 2.4. Molecular Detection of *P. mirabilis* Virulence Genes

Several virulence genes were detected using conventional PCR amplification. The PCR cycling protocol and primer sequences for each gene were previously described [11, 17–20]. Confirmation of gene identity relied on finding a band corresponding to expected PCR product size.

### 2.5. Statistical Analysis

For the present study, statistical analysis of data using appropriate programs and methods such as the Statistical Package for the Social Sciences (SPSS) version 23 was performed to generate descriptive analysis of raw data, including generation of all frequency tables and cross tabulations. The Pearson Chi-squared test was used to compare frequency data. *P* value less than 0.05 was considered statistically significant.

## 3. Results

### 3.1. Antimicrobial Susceptibility Results

The antimicrobial susceptibility results of the *P. mirabilis* isolates to several antimicrobial agents are shown in Table 1, which represents results as susceptible, intermediate, and resistant. The rate of antibiotic resistance was highest for nitrofurantoin (88.9%), whereas it was lowest for ceftriaxone (1.6%).

### 3.2. *P*. *mirabilis* Urovirulence Genes

Detected rates of virulence genes are shown in Table 2. The antimicrobial susceptibility was highly correlated with the presence of *P. mirabilis* virulence genes (Table 3). Moreover, *hpmA*, *ureC1*, *rpoA*, *atfC*, *mrpA*, and *pm1* urovirulence genes were more likely to coexist with each other at *P. mirabilis* (*P* < 0.001).

## 4. Discussion

In the current study, *P. mirabilis* isolated from UTI patients were analyzed for the presence of virulence genes and susceptibility to antimicrobials. *P. mirabilis* genes associated with UTIs may be valuable in developing strategies for treating and preventing UTIs. The results of this study provide evidence supporting the role of urovirulence genes of *P. mirabilis* in human UTIs.

Current results showed the association between resistance to certain antibiotics and the presence of *P. mirabilis* urovirulence genes. For example, *ureC1* and *atfC* genes were associated with resistance to cephalexin, *atfA* with resistance to sulfamethoxazole, *hpmA*, *ureC1*, *rpoA*, *atfC*, *mrpA*, and *pm1* with resistance to ceftriaxone, and *hpmA*, *ureC1*, *rpoA*, *atfA*, *atfC*, *mrpA*, and *pm1* with resistance to nitrofurantoin.

The correlation between presence of these genes and the increase in resistance toward antibiotics may be attributed to pathogenicity of these genes and their functional roles such as urease, hemolysins, and fimbriae that help the organism to overcome host defense mechanisms and colonize the urinary tract. Overall, these results explain the potential of these uropathogens to interfere with the infection treatment, impair the action of host immune cells, and weaken the antibiotic efficiency.

Most isolates were resistant to nitrofurantoin (88.9%), ampicillin (61.9%), and sulfamethoxazole (55.6%). On the contrary, the highest sensitivity was against ceftriaxone (96.8%), norfloxacin (82.5%), gentamicin (71.4%), and ciprofloxacin (69.8%). Similar results have been reported for *P. mirabilis* from Nigeria, where isolates' resistance rates to ciprofloxacin, nalidixic acid, sulfamethoxazole, and gentamicin were 13.9%, 53.7%, 74.1%, and 26.9%, respectively [21]. In Czech Republic, the isolates had a resistance rate of ciprofloxacin (35.2%), sulfamethoxazole (39.0%), ampicillin (38.5%), and gentamicin (25.4%), which are different form findings of the present study [22]. The noticed variations in resistance rates may be referred to regional variation in bacterial strain and virulence genes prevalence, in addition to different standards and controls for prescription and use of antimicrobial agents.

Urovirulence genes of *P. mirabilis* strains are poorly identified among UTI patients. One of the aims of this study was to identify the urovirulence genes of *P. mirabilis* strains isolated from UTI symptomatic patients. Specifically, we investigated the presence of urovirulence genes *hpmA*, *hpmB*, *rsbA*, *luxS*, *ureC1*, *hlyA*, *rpoA*, *atfA*, *atfC*, *mrpA*, and *pm1* using PCR-based analysis. Certain patterns of virulence genes and distributions were identified among the isolates. Statistically significant associations were observed among the *P. mirabilis* urovirulence genes, as some genes were more likely to coexist with other genes. There was coassociation between *hpmA*, *ureC1*, *rpoA*, *atfC*, *mrpA*, and *pm1* genes. Therefore, it is likely that a frequent occurrence of antimicrobial resistance is due to the presence of multiple resistance genes that increase the *P. mirabilis* pathogenicity.

Virulence genes were detected at the following rates among the isolates: *hpmB*, *rsbA*, and *luxS* at 100%, *hpmA* and *atfA* at 98.4%, *rpoA* at 96.8%, *ureC1* and *atfC* at 95.2%, *mrpA* and *pm1* at 92.1%, and *hlyA* at 23.8%. Some of these prevalence rates are different from those reported from other countries [4, 11, 22]. Prevalence of these genes may vary according to the clinical status of the host and the genetic makeup of the isolates causing UTIs. The *hpmB*, *luxS*, and *rsbA* genes were the most prevalent at 100%, followed by *hpmA* and *atfA* at 98.4% each, while the *hlyA* gene was the least prevalent at 23.8%. Other urovirulence genes were prevalent in 92–97% of the isolates. Additionally, the high prevalence of *hpmB* and *hpmA* at 100% and 98.4%, respectively, in the present study was consistent with a previous report from Brazil [11]. On the contrary, the prevalence of *hlyA* (23.8%) is different from the same study, which confirmed that none of the isolates presented *hlyA* gene [11]. Interestingly, another study from Iraq reported *ureC1*, *mrpA*, *pm1*, *luxS*, and *rsbA* prevalence rates of 18%, 35%, 41%, 47%, and 53%, respectively, which are not comparable to our findings [4], whereas a study from Iran reported *luxS*, and *rsbA* prevalence rate of 70% each [17]. The previously mentioned prevalence rates most likely attribute to the differences in the distribution of virulence genes among different populations and geographic locations. The current study has some limitations including that it tested only certain virulence genes and certain antibiotics. Studying more virulence genes and antibiotics is recommended future study.

None of the previous studies have investigated a role of *atfA* and *atfC* urovirulence genes in *P. mirabilis* UTIs. Current results suggest that *ATF* fimbriae could have an important role in adhesion and biofilm formation on abiotic [20, 23].

In conclusion, *P. mirabilis* isolates demonstrated high susceptibility against ceftriaxone, norfloxacin, gentamicin, and ciprofloxacin, and high resistance against nitrofurantoin, ampicillin, and sulfamethoxazole. In addition, significant associations between virulence genes and resistance phenotypes were identified, which suggests increased resistance to antimicrobial agents due to the presence of these virulence genes.

## Figures and Tables

**Table 1:** Antimicrobial susceptibility of *P. mirabilis*.

Antimicrobial agent	Susceptible (%)	Intermediate (%)	Resistant (%)
Ciprofloxacin	44 (69.8%)	11 (17.5%)	8 (12.7%)
Cephalexin	33 (52.4%)	9 (14.3%)	21 (33.3%)
Nalidixic acid	27 (42.9%)	7 (11.1%)	29 (46%)
Sulfamethoxazole	25 (39.7%)	3 (4.8%)	35 (55.6%)
Ceftriaxone	61 (96.8%)	1 (1.6%)	1 (1.6%)
Nitrofurantoin	2 (3.2%)	5 (7.9%)	56 (88.9%)
Norfloxacin	52 (82.5%)	4 (6.3%)	7 (11.1%)
Ampicillin	23 (36.5%)	1 (1.6%)	39 (61.9%)
Gentamicin	45 (71.4%)	5 (7.9%)	13 (20.6%)

**Table 2:** Frequency of urovirulence genes among the isolates of *P. mirabilis*.

Gene	Present (%)
hpmA	62 (98.4%)
hpmB	63 (100%)
rsbA	63 (100%)
luxS	63 (100%)
ureC1	60 (95.2%)
hlyA	15 (23.8%)
rpoA	61 (96.8%)
atfA	62 (98.4%)
atfC	60 (95.2%)
mrpA	58 (92.1%)
pm1	58 (92.1%)

**Table 3:** Association between urovirulence genes and susceptibility to antimicrobial agents in *P. mirabilis* isolates.

Antibiotics/genes		CIP			CL			NA			SMX			CTX			NFT			NFX			AMP			GN		
*hpmA*		S	I	R	S	I	R	S	I	R	S	I	R	S	I	R	S	I	R	S	I	R	S	I	R	S	I	R
	−	1	0	0	0	0	1	0	0	1	0	0	1	0	0	1	1	0	0	1	0	0	0	0	1	1	0	0
	+	43	11	8	33	9	20	27	7	28	25	3	34	61	1	0	1	5	56	51	4	7	23	1	38	44	5	13
	*P* value	0.803			0.362			0.551			0.666			**0.000**			0.000			0.898			0.732			0.816		
*hpmB*	−	0	0	0	0	0	0	0	0	0	0	0	0	0	0	0	0	0	0	0	0	0	0	0	0	0	0	0
	+	44	11	8	33	9	21	27	7	29	25	3	35	61	1	1	2	5	56	52	4	7	23	1	39	45	5	13
	*P* value	—			—			—			—			—			—			—			—			—		
*rsbA*	−	0	0	0	0	0	0	0	0	0	0	0	0	0	0	0	0	0	0	0	0	0	0	0	0	0	0	0
	+	44	11	8	33	9	21	27	7	29	25	3	35	61	1	1	2	5	56	52	4	7	23	1	39	45	5	13
	*P* value	—			—			—			—			—			—			—			—			—		
*luxS*	−	0	0	0	0	0	0	0	0	0	0	0	0	0	0	0	0	0	0	0	0	0	0	0	0	0	0	0
	+	44	11	8	33	9	21	27	7	29	25	3	35	61	1	1	2	5	56	52	4	7	23	1	39	45	5	13
	*P* value	—			—			—			—			—			—			—			—			—		
*ureC1*	−	1	2	0	0	0	3	0	0	3	1	0	2	2	0	1	1	2	0	3	0	0	0	0	3	3	0	0
	+	43	9	8	33	9	18	27	7	26	24	3	33	59	1	0	1	3	56	49	4	7	23	1	36	42	5	13
	*P* value	0.068			**0.043**			0.158			0.882			**0.000**			0.000			0.717			0.379			0.533		
*hlyA*	−	34	9	5	22	9	17	18	6	24	18	2	28	47	0	1	2	4	42	39	4	5	18	1	29	33	4	11
	+	10	2	3	11	0	4	9	1	5	7	1	7	14	1	0	0	1	14	13	0	2	5	0	10	12	1	2
	*P* value	0.592			0.094			0.303			0.715			0.171			0.702			0.502			0.803			0.687		
*rpoA*	−	1	1	0	0	0	2	0	0	2	1	0	1	1	0	1	1	1	0	2	0	0	0	0	2	2	0	0
	+	43	10	8	33	9	19	27	7	27	24	3	34	60	1	0	1	4	56	50	4	7	23	1	37	43	5	13
	*P* value	0.442			0.127			0.298			0.921			**0.000**			0.000			0.804			0.530			0.662		
*atfA*	−	1	0	0	0	0	1	0	0	1	0	1	0	1	0	0	1	0	0	1	0	0	0	0	1	1	0	0
	+	43	11	8	33	9	20	26	7	29	25	2	35	60	1	1	1	5	56	51	4	7	23	1	38	44	5	13
	*P* value	0.803			0.362			0.508			**0.000**			0.983			0.000			0.898			0.732			0.816		
*atfC*	−	1	2	0	0	0	3	0	0	3	1	0	2	2	0	1	1	2	0	3	0	0	0	0	3	3	0	0
	+	43	9	8	33	9	18	27	7	26	24	3	33	59	1	0	1	3	56	49	4	7	23	1	36	42	5	13
	*P* value	0.068			**0.043**			0.158			0.882			**0.000**			0.000			0.717			0.379			0.533		
*mrpA*	−	3	1	1	1	1	3	0	1	4	2	1	2	4	0	1	1	1	3	4	0	1	1	0	4	4	0	1
	+	41	10	7	32	8	18	27	6	25	23	2	33	57	1	0	1	4	53	48	4	6	22	1	35	41	5	12
	*P* value	0.851			0.306			0.130			0.236			**0.003**			0.042			0.692			0.677			0.784		
*pm1*	−	2	2	1	2	2	1	0	1	4	2	0	3	4	0	1	1	1	3	4	0	1	2	0	3	4	0	1
	+	42	9	7	31	8	19	27	6	25	23	3	32	57	1	0	1	4	53	48	4	6	21	1	36	41	5	12
	*P* value	0.286			0.837			0.130			0.870			**0.003**			0.042			0.692			0.948			0.784		

Ciprofloxacin: CIP, cephalexin: CL, nalidixic acid: NA, sulfamethoxazole: SMX, ceftriaxone: CTX, nitrofurantoin: NFT, norfloxacin: NFX, ampicillin: AMP, and gentamicin: GN.

## Data Availability

The data used to support the findings of this study are available from the corresponding author upon request.
